# Supplementary figures and images for: Diagnostic accuracy of three computer-aided detection systems for detecting pulmonary tuberculosis on chest radiography when used for screening: Analysis of an international, multicenter migrants screening study
Source: PLOS Glob Public Health. 2023 Jul 14;3(7):e0000402. doi: 10.1371/journal.pgph.0000402 (PMC10348531; doi:10.1371/journal.pgph.0000402)

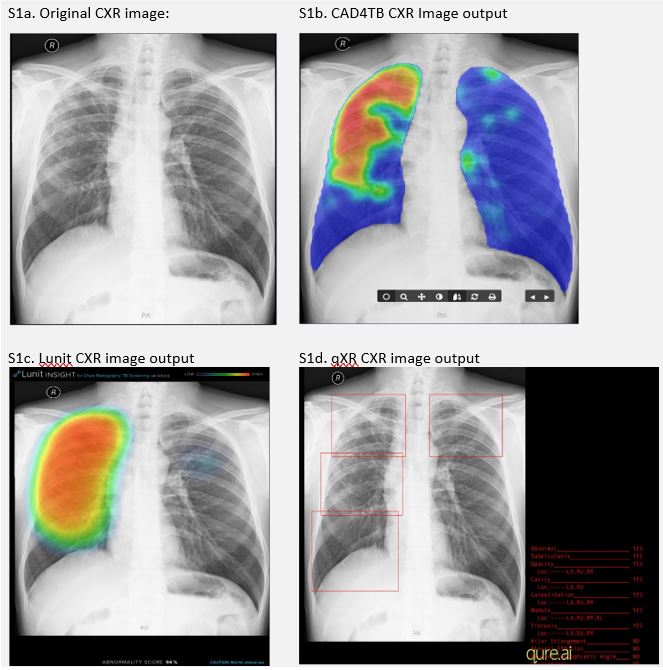

Supplement: S1 Fig — A Sample chest x-ray image before and after the image processing by each CAD system, with image output heat maps/ boxes, S1A–S1D Fig. CAD abnormality scores were 81 for CAD4TB, 94% for Lunit, and 0.86 for qXR. The CXR finding of the case was suggestive of TB and culture positive. (TIF) [file pgph.0000402.s008.tif]

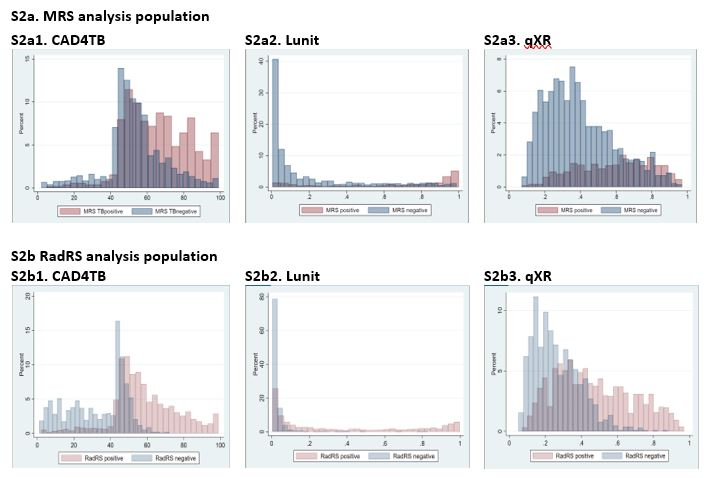

Supplement: S2 Fig — Two-way histogram distribution of abnormality scores from three CAD systems for a) MRS analysis population, and b) RadRS analysis population. (TIF) [file pgph.0000402.s009.tif]

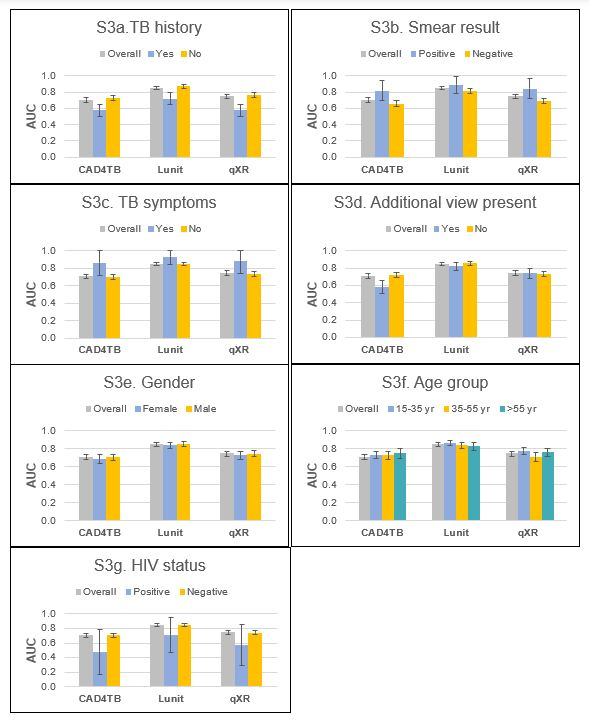

Supplement: S3 Fig — Diagnostic accuracy of three CAD systems across population subgroups, S3A-S3G Fig. (TIF) [file pgph.0000402.s010.tif]
